# Social Selection and Indirect Genetic Effects in Structured populations

**DOI:** 10.1007/s11692-013-9252-5

**Published:** 2013-08-11

**Authors:** Barbora Trubenová, Reinmar Hager

**Affiliations:** Computational and Evolutionary Biology, Faculty of Life Sciences, The University of Manchester, Michael Smith Building, Oxford Road, Manchester, M13 9PT UK

**Keywords:** Indirect genetic effect, Direct genetic effect, Social interaction, Social selection

## Abstract

Social selection and indirect genetic effects (IGEs) are established concepts in both behavioural ecology and evolutionary genetics. While IGEs describe effects of an individual’s genotype on phenotypes of social partners (and may thus affect their fitness indirectly), the concept of social selection assumes that a given phenotype in one individual affects the fitness of other individuals directly. Although different frameworks, both have been used to investigate the evolution of social traits, such as cooperative behaviour. Despite their similarities (both concepts consider interactions among individuals), they differ in the type of interaction. It remains unclear whether the two concepts make the same predictions about evolutionary trajectories or not. To address this question, we investigate four possible scenarios of social interactions and compare the effects of IGEs and social selection for trait evolution in a multi-trait multi-member model. We show that the two mechanisms can yield similar evolutionary outcomes and that both can create selection pressure at the group level. However, the effect of IGEs can be stronger due to the possibility of feedback loops. Finally, we demonstrate that IGEs, but not social selection gradients, may lead to differences in the direction of evolutionary response between genotypes and phenotypes.

## Introduction

Social interactions are common in nature and have profound effects on evolutionary processes (West-Eberhard and Rica 1979). Understanding the role and effects of interactions among individuals for phenotypic variation and fitness is central to research in such diverse areas as behavioural ecology (Trivers 1974; Moore et al. 2002; Davies et al. 2012) or quantitative genetics (Wolf et al. 1998; Cheverud 2003; Kölliker et al. 2005), for example for generating predictions about evolutionary trajectories and patterns of past selection.

Any interaction between individuals that influences the fitness of other individuals can be regarded as social (Wilson and Wilson 2007). For example, cooperation, altruism, but also aggression, spite and dominance are social behaviours, and traits underlying these behaviours are influenced by interactions between individuals (Gardner and West 2004; West et al. 2007b; Wilson et al. 2009). Indeed, interactions that create the social environment are often the most important component of the environment and can thus have profound effects on trait expression, fitness and evolution (Wolf 2003).

**Fig. 1:**
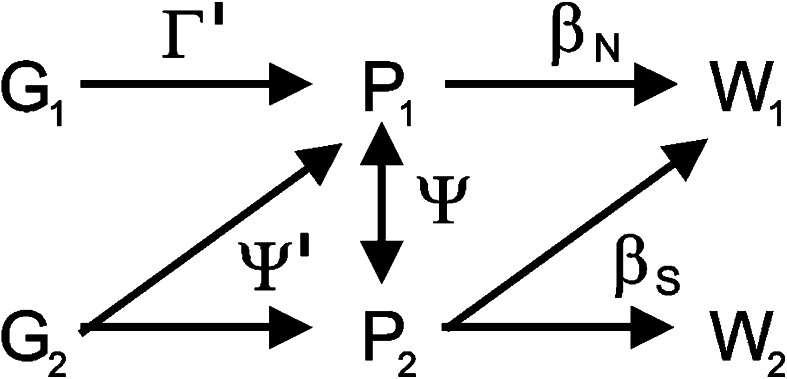
Different types of social interactions. Indirect genetic effects ($\varPsi^\prime$) describe effects of other genotypes on the phenotype of a focal individual, while $\varGamma^\prime$ describes direct genetic effects. Interactions between phenotypes are described by $\varPsi.$ The social selection gradient β_*S*_ describes direct effects of phenotypes on fitness of conspecifics, while β_*N*_ denotes the non-social selection gradient. *G* represents genotype, *P* phenotypes and *W* absolute fitness of each individual

Individuals can be affected by two different types of social interactions, those that affect an individual’s phenotype via indirect genetic effects (IGEs) and those that affect individual fitness only via a social selection gradient (Fig. 1). On the one hand, social selection models assume that a trait in one individual directly influences the fitness of its social partners, for example, vigilance behaviour shown by one individual may affect the fitness of other individuals in the group (West-Eberhard and Rica 1979; West-Eberhard 1983; Frank 1997; Wolf et al. 1999; Bijma and Wade 2008). These influences are usually described by a social selection gradient (Queller 1992; Wolf et al. 1999; Agrawal 2001; Bijma and Wade 2008; McGlothlin et al. 2010; Westneat 2011). For example, kin selection is a type of social selection and plays a key role in the evolution of social traits that are not easily explained by non-social selection, such as altruism (Hamilton 1963, 1964a, b; Agrawal 2001) or spite (West et al. 2001; Gardner and West 2006, Gardner and West 2010).

On the other hand, some traits involved in social interactions do not affect the fitness of conspecifics directly but may affect the expression of other traits in social partners. For example, scent marks that signal territoriality to competitors may alter the competitor’s behaviour toward the signaller, e.g. in rodents (Hurst and Beynon 2004). Therefore, an individual’s phenotype can be affected by phenotypes of other individuals, which, in turn, are influenced by their respective genotypes. The influence of the genotypes of other individuals on the phenotype of a focal individual is referred to as associative (Muir 2005; Hadfield and Wilson 2007; Bijma 2010a) or indirect genetic effect (Moore et al. 1997; Wolf et al. 1998; Wolf 2000; Agrawal et al. 2001; McGlothlin and Brodie III 2009; Wilson et al. 2009; Bijma 2010b; Teplitsky et al., 2010); a given trait in a focal individual is influenced indirectly by genes expressed in social partners.

In contrast to effects of the physical environment, IGEs have both an environmental and genetic (thus heritable) component and can therefore be subject to selection and subsequent evolution. Because IGEs are part of the environment individuals experience, they can change the strength of selection as well as the expected genotype-phenotype relationship, and thus the speed and direction of evolution (Moore et al. 1997; Wolf et al. 1998; McGlothlin et al. 2010). Moreover, IGEs enable evolutionary change in traits that have no direct additive genetic variance (Moore et al. 1997; Wolf et al. 1998; Wolf 2000).

Social selection and IGE models have been used, both jointly and in separate treatments, to explain trait evolution and social behaviour (Wolf et al. 1999; Bijma and Wade 2008; Agrawal 2001; McGlothlin et al. 2010). The main distinction between the two is whether or not there is a direct effect of a particular trait on the fitness of the social partner. Direct effects on fitness are described by the social selection gradient, while IGEs capture the effect on phenotypes of conspecifics, which may or may not influence their fitness indirectly.

Wolf et al. (1999) developed a model of social selection of interacting phenotypes that can be evaluated independently from the genetics of interacting phenotypes. This model showed that an opportunity for social selection exists whenever individual fitness varies as a result of interactions with conspecifics (Wolf et al. 1999). Thus, in this context, IGEs are one of many possible factors that may contribute to the covariance of interacting phenotypes.

Further, Agrawal (2001) pointed out the importance of population structure when considering social interactions and showed that non-linear social effects in subdivided populations alter the evolutionary response that may lead to the opposite of what would be expected due to direct selection. However, the authors did not investigate interactions where one trait affects its own expression through interaction with other traits in social partners, which leads to a feedback loop (Wolf et al. 1999; McGlothlin et al. 2010) and may cause extreme phenotypes (Trubenová and Hager 2012). For example, aggressive behaviour by one individual may increase aggression in another individual, which will lead to elevated levels of aggression in all members of the group. Furthermore, small differences between individual genotypes may have strong effects on the phenotypes of all group members. In scenarios where feedback loops occur, group size can be very important in determining levels of trait expression (e.g. levels of aggression). For example, while a group of a given size may be relatively stable because the level of aggression is sufficiently low, a small deviation from this group size (e.g. a new group member) may cause a dramatic increase in within-group aggression such that groups become unstable and break up (Trubenová and Hager 2012). We consider the possibility of feedback loops caused by IGEs to be one of the key distinguishing features between the two types of interactions (IGEs and social selection).

Later, Bijma and Wade (2008) also integrated both social selection and IGE models and investigated the effects of multilevel selection, IGEs and relatedness on response to selection in a single trait model. The authors concluded that the response to selection depends both on relatedness and the degree of multilevel selection. Bijma and Wade (2008) showed that when IGEs are present multilevel selection can explain the evolution of social traits, even in the absence of relatedness among individuals. Moreover, the authors pointed out that IGEs can lead to social selection in the absence of a social selection gradient.

Finally, McGlothlin et al. (2010) analysed the role of IGEs in social and non-social selection. The authors concluded that social selection can lead to evolution only if traits expressed by social partners are non-randomly associated, for example in family groups where individuals are more likely to interact with other family members than with non-family members. McGlothlin et al. (2010) also discussed the possibility of feedback loops when one trait affects the expression of the same trait in social partners. Later, Westneat (2011) expanded on McGlothlin’s model by incorporating interactive effects of conspecific traits on the fitness of the focal individual.

Here, we develop an analytical, multi-trait model supported by agent-based modelling of multiple interacting individuals. We investigate and compare the effect of social selection and IGEs on the evolution of social traits and highlight similarities and differences between both concepts. We analyse four scenarios in which either IGEs or a social selection gradient occurs, both of these, or neither. In all of these scenarios, non-social selection occurs. Subdividing the population of interacting individuals into smaller groups was shown to have crucial consequences for the evolution of social traits such as cooperation (Agrawal 2001). Therefore, we partitioned the population of individuals into *M* groups of *N* interacting individuals. However, in contrast to Agrawal (2001), we use a more general model of interaction that involves the possibility of feedback loops, when a trait (e.g. aggression) has an effect on expression of the same trait in social partners [as in Wolf et al. (1999); McGlothlin et al. (2010); Trubenová and Hager (2012)]. We show that such feedback loops are the major difference between IGEs and social selection models because they may cause a difference between the direction of phenotypic and genotypic evolution.

## The Model

To investigate differences between the effects of social selection and IGEs for the evolution of sociality, we model four different scenarios in a structured population, which consists of *M* groups of *N* interacting individuals. While individuals within each group interact with each other, there is no interaction between members of different groups. The four possible scenarios are: (1) no IGEs occur and no social selection gradient exists, (2) no IGEs occur but a social selection gradient exists, (3) IGEs do occur but no social selection gradient exists, and (4) both IGEs and social selection gradient exist. In all scenarios, non-social selection occurs. For each scenario, we derive equations for the relative fitness of individuals and the response to selection as a change of the mean genotypic value between the two subsequent, non-overlapping generations. The relative fitness of an individual is given by the ratio of total number of its offspring over the total number of offspring in the next generation.

In standard quantitative genetic models, the phenotype of an individual is given by

1 $$ {\user2{p}} = {\varvec{\varGamma}} \user2{g} + \user2{e} $$

where matrix ${\varvec{\varGamma}}$ translates an individual’s own genotype described by the column vector ${\user2{g}}$ into its corresponding trait values, described by column vector ${\user2{p}}.$ For the simplest models, where every trait is encoded by a single gene, this matrix is diagonal. However, in a more realistic scenario, where multiple genes affect the same trait or the same gene affects more than one trait (pleiotropy), the matrix $\varvec{\varGamma}$ is more populated. Vector ${\user2{e}}$ captures (abiotic) environmental influences. For simplicity, we assume that the effects of the abiotic environment on individual phenotypes are negligible. Therefore, ${\user2{e}} = {\bf 0}$ in our model. The mean genotype in the whole parental population is set to 0 $(\overline{\overline{{\user2{g}}}} = {\bf 0}).$

Non-social selection is represented by a gradient given by the row vector $\varvec{\beta}_{\user2{N}}$ that quantifies the strength of selection on each trait (Lande 1979; Lande and Arnold 1983). The social selection gradient is represented by a row vector ${\varvec{\beta}}_{\user2{S}}$ describing the effects of trait values of other individuals on the focal individual’s fitness.

The response to selection ${\varvec{\vartriangle}}{\overline{\overline{\user2{g}}}}$ is defined as the difference between the mean offspring genotype ${\overline{\overline{\user2{g}}}}_{\user2{o}}$ and the parental mean genotype ${\overline{\overline{\user2{g}}}}_{\user2{p}},$ and can be calculated using the Price equation, assuming perfect transmission (Price 1970; Frank 1995; Gardner 2008):

2 $$ \varvec{\vartriangle} {\overline{\overline{\user2{g}}}} = \overline{\overline{\user2{g}}}_{\user2{o}}-\overline{\overline{\user2{g}}}_{\user2{p}} = MN cov (w, {\user2{g}}) $$

where $MNcov (w, {\user2{g}})$ is the covariance of fitness and genotypes multiplied by the total number of all individuals.

We assume that the mean fitness of the population is 1/*MN* (Frank 1997) in contrast to 1 as often used elsewhere. We use this value so that the sum of all fitness across the population is 1, which simplifies the model.

## Results

### No IGEs Occur and No Social Selection Gradient Exists

In the simplest scenario, the phenotype of a focal individual depends only on its own genotype and the non-social environment. Thus, no IGEs and no social selection gradient exists. Even if individuals interact, their phenotypes and fitness are unaffected by these interactions.

Absolute fitness *W* of an individual is given by

3 $$ W_{ki}={\varvec{\beta}}_{\user2{N}} {\user2{p}}_{\user2{ki}} + C = \varvec{\beta}_{\user2{N}} \varvec{\varGamma} {\user2{g}}_{\user2{ki}} + C $$

where ${\user2{p}}_{\user2{ki}} ({\user2{g}}_{\user2{ki}})$ is a column vector describing the phenotype (genotype) of the *ith* individual in the *kth* group, ${\varvec{\beta}}_{\user2{N}}$ is a row vector describing the effect of trait values on fitness (non-social selection gradient, as in Lande and Arnold (1983); Frank (1997); Wolf et al. (1999); McGlothlin et al. (2010)) and *C* is a positive constant.

If we standardize phenotypic values, such that the mean phenotype of the population ${\overline{\overline{\user2{p}}}} = {\bf 0},$ the relative fitness can be calculated and is given by

4 $$ w_{ki} = \frac{{\varvec{\beta}}_{\user2{N}} {\user2{p}}_{\user2{ki}}+ C}{MNC} $$

5 $$ = \frac{{\varvec{\beta}}_{\user2{N}}{\varvec{\varGamma}} {\user2{g}}_{\user2{ki}}+ C}{MNC} $$

In this case the relative fitness of a particular individual depends linearly on its own trait values.

The response to selection is given by

6 $$ \varvec{\vartriangle} \overline{\overline{\bf g}}^{T} = \frac{\varvec{\beta}_{\user2{N}} \varvec{\varGamma} \user2{C}_{\user2{g}}}{C} $$

where $C_{\user2{g}}$ is a covariance matrix of all genetic values. Diagonal elements of the matrix are genotypic variances of all traits while non-diagonal elements represent the covariance between genetic values and will result in a correlated response to selection (Lande 1979).

Equation (6) is equivalent to those derived by Lande (1979) and Lande and Arnold (1983) although here we express the change in mean genotype, not phenotype. In this first scenario, the response to selection is unaffected by population structure. Therefore, if only non-social selection acts on the population, with no IGEs present, the population structure does neither affect the direction nor the rate of trait evolution.

### No IGEs Occur but a Social Selection Gradient Exists

It has long been recognized that social selection enables the evolution of traits that would not evolve and persist under conditions of non-social selection, such as altruism or spite (Haldane 1932; Hamilton 1963; Maynard Smith 1964; Frank 1997; Gardner and West 2010). In this second scenario, the fitness of each individual is directly affected by traits expressed in its social partners but phenotypes are not altered by the interaction, i.e. no IGEs occur. An example of such a situation is cooperative defence, where individuals help protect offspring other than own young, e.g. in red-winged blackbirds (Olendorf et al. 2004). While offspring fitness may be affected through protection, specific offspring traits in the focal individual are not necessarily affected.

Here, the relative fitness of an individual is given by

7 $$ w_{ki}= \frac{(\varvec{\beta}_{\user2{N}} + (\user2{N}-{\bf 1})\varvec{\beta}_{\user2{S}})\varvec{\varGamma} \overline{\user2{g}}_{\user2{k}}+(\varvec{\beta}_{\user2{N}} -\varvec{\beta}_{\user2{S}}){\varvec{\varGamma}} {\varvec{\Updelta}} \user2{g}_{\user2{ki}} + C}{MNC} $$

where $\varvec{\beta}_{\user2{S}}$ denotes a row vector describing the direct effect of social partner traits on the fitness of the focal individual [social selection gradient; Queller (1992); Wolf et al. (1999); Agrawal (2001); Bijma and Wade (2008); McGlothlin et al. (2010); Westneat (2011)], ${\overline{\user2{g}}}_{\user2{k}}$ is a vector of the mean genotype in the *kth* group and ${\varvec{\Updelta}} \user2{g}_{\user2{ki}}$ is the deviation of an individual’s genotype from the mean of its group.

In this second scenario, it can be seen that if a trait increases absolute fitness of an individual’s social partners, but has no effect on the absolute fitness of the bearer, this trait will decrease the relative fitness of its bearer. However, if a trait increases both the focal individual and its social partners’ absolute fitness, it is no longer obvious whether the trait increases or decreases relative fitness of the focal individual. In contrast to the first scenario, here the response to selection (and therefore the direction of evolution) will depend on the structure of the population.

Traditionally, the response to selection is calculated using the variance (or heritability expressed as the ratio of variances) of a particular trait in a population. However, because we model social interactions, group properties are important and we will express the response to selection using intra- and inter-group variance (inter- and intra-group genotypic variance-covariance matrix in case of multiple genes).

The response to selection is given by

8 $$ \vartriangle\overline{\overline{\varvec{g}}}^T =\frac{(\varvec{\beta}_{\user2{N}}+(N-1){\varvec{\beta}_{\user2{S}}}) \varvec{\varGamma}{\user2{C}}_{{\overline{\user2{g}}}} +({\varvec{\beta}}_{\user2{N}}-\varvec{\beta}_{\user2{S}})\varvec{\varGamma} {\user2{C}_{\varvec{\vartriangle} \user2{g}}}}{C} $$

where ${\user2{C}}_{\overline{\user2{g}}}$ is the variance-covariance matrix of mean group genotype (between group variance-covariance; first part of the equation), and ${\user2{C}}_{\varvec{\vartriangle}\user2{g}}$ is a variance-covariance matrix of genotypes within the group (within group variance-covariance; second part of the equation). In our notation, a double over-line denotes the population average, while a single over-line refers to the group mean.

Note the importance of the group structure. Equation (8) shows that the response to selection depends not only on the overall genotypic variance of the population, but also on the intra-group and inter-group genotypic variances as well. The ratio between intra and inter-group genotypic variance determines whether the response to selection is positive or negative.

When simplified, Eq. (8) agrees with Queller (1992) who modelled a similar scenario and showed that the response to selection can be partitioned in between and within-group parts, each with its own selection differential and heritability (assumed to be 1 in our model).

### IGEs Occur but No Social Selection Gradient Exists

Some interactions between individuals may have no direct consequences for individual fitness, however, they may still affect trait expression, in which case IGEs occur but no social selection gradient. For example, individuals may adjust their aggressiveness in relation to the size of their social partners (Brenner et al. 1978; Thornhill 1984; Huntingford and Turner 1987). Here, an individual’s phenotype is affected by the expression of genes in its social partners, however, there is no direct effect on their fitness, i.e. no social selection gradient is present.

The phenotype of the focal individual in a group of *N* individuals is given by

9 $$ \user2{p}_{\user2{ki}} = \varvec{\varGamma} \user2{g}_{\user2{ki}} + \sum_{j \neq i}^{N-1} \varvec{\varPsi} \user2{p}_{\user2{kj}} $$

where matrix $\varvec{\varPsi}$ is a square (*m* × *m*) interaction matrix (Moore et al. 1997; Wolf et al. 1998; McGlothlin et al. 2010), in which $\varPsi_{kl}$ defines the effect of the partner’s trait *l* on trait *k* of the focal individual. If $\varPsi_{kl}$ equals 0, there is no effect, if it is negative, a higher expression of the partner’s trait *l* lowers the expression of the focal individual’s trait *k*. A positive $\varPsi_{kl}$ means that the expression of trait *l* enhances the expression of trait *k* in the focal individual. *N* denotes the number of individuals in the group, *i* the focal individual and *j* all other individuals in the group.

To separate the effects of an individual’s own genes from those of its social partners, we can rewrite Eq. (9) as follows

10 $$ \begin{aligned} {\user2{p}}_{\user2{i}} & = \underbrace{(\user2{I}+\varvec{\varPsi})^{-1} (\user2{I}+ \varvec{\varPsi} ( \user2{I} - N \varvec{\varPsi} + \varvec{\varPsi} )^{-1}) \varvec{\varGamma} }_{\varvec{\varGamma}'}\user2{g}_{\user2{i}}\\ &+ \underbrace{(\user2{I}+ \varvec{\varPsi})^{-1} \varvec{\varPsi} (\user2{I} - N \varvec{\varPsi}+ \varvec{\varPsi})^{-1} \varvec{\varGamma}}_{\varvec{\varPsi}^{\varvec{\prime}}} \sum_{j \neq i}^{N-1} \user2{g}_{\user2{j}} \end{aligned} $$

where $\user2{I}$ is an identity matrix, $\varvec{\varGamma}^{\varvec{\prime}}$ is a matrix of direct genetic effects and $\varvec{\varPsi}^{\varvec{\prime}}$ is a matrix of indirect genetic effects (Trubenová and Hager 2012).

As we assume no social selection gradient, the relative fitness of an individual is given by Eq. (4). By substituting Eq. (10) into Eq. (4), we can express relative fitness as

11 $$ w_{ki}={\frac{\varvec{\beta}_{\user2{N}}(\varvec{\varGamma}^{\varvec{\prime}}+ (N-1)\varvec{\varPsi}^{\varvec{\prime}}) {\overline{\user2{g}}}_{\user2{k}} + \varvec{\beta}_{\user2{N}}(\varvec{\varGamma}^{\varvec{\prime}}-\varvec{\varPsi}^{\varvec{\prime}}) \varvec{\Updelta} \user2{g}_{\user2{kj}}+C}{NMC}} $$

and the response to selection is given by

12 $$ \vartriangle\overline{\overline{\user2{g}}}^T ={\frac{\varvec{\beta}_{\user2{N}}(\varvec{\varGamma}^{\varvec{\prime}} +(N-1)\varvec{\varPsi}^{\varvec{\prime}})\user2{C}_{\overline{\user2{g}}} +\varvec{\beta}_{\user2{N}}(\varvec{\varGamma}^{\varvec{\prime}}-\varvec{\varPsi}^{\varvec{\prime}}) \user2{C}_{\varvec{\vartriangle} \user2{g}}}{C}}. $$

Equation (12) shows that the strength of the indirect genetic effect given by $\varvec{\varPsi}$ is a key factor determining which of the variances (intra- or inter-group) is more important for the response to selection.

While in the previous (second) case the importance of the inter-group variance was multiplied by social selection with the coefficient *N* − 1 (number of social partners), in this scenario, it is IGEs that determine the importance of the inter-group variance, again with the coefficient (*N* − 1). Further, the intra-group variance is multiplied by a difference between DGEs and IGEs $(\varvec{\varGamma}^{\varvec{\prime}} -\varvec{\varPsi}^{\varvec{\prime}}),$ whereas in the second scenario it was the difference between non-social and social selection gradients $(\varvec{\beta}_{\user2{N}} -\varvec{\beta}_{\user2{S}})$.

### Both IGEs and Social Selection Gradient Exists

In the last scenario, both IGEs and social selection gradient are present. The interactions among individuals affect the fitness of all interactants directly, as well as their phenotypic trait values. For example, maternal care can affect both fitness directly (by protecting young), or influence the expression of traits in her offspring (such as body size affected by provisioning), which may influence their fitness indirectly.

Here, the fitness of the *ith* individual in the *kth* group is given by

13 $$ \begin{aligned} w_{ki} &= {\frac{(\varvec{\beta}_{\user2{N}}+ (N-1) \varvec{\beta}_{\user2{S}})(\varvec{\varGamma}^{\varvec{\prime}} +(N-1)\varvec{\varPsi}^{\varvec{\prime}}) {\overline{\user2{g}}}_{\user2{k}}}{NMC}}\\ & +{\frac{(\varvec{\beta}_{\user2{N}}-{\varvec{\beta}_{\user2{S}}}) (\varvec{\varGamma}^{\varvec{\prime}}-\varvec{\varPsi}^{\varvec{\prime}}) \vartriangle \user2{g}_{\user2{ki}} +C}{ NMC }} \end{aligned} $$

and the response to selection by

14 $$ \begin{aligned} \vartriangle\overline{\overline{\user2{g}}}^T &={\frac{[(\varvec{\beta}_{\user2{N}}+(N-1)\varvec{\beta}_{\user2{S}}) (\varvec{\varGamma}^{\varvec{\prime}}+ (N-1)\varvec{\varPsi}')\user2{C}_{\overline{\user2{g}}}}{C}}\\ &+{\frac{(\varvec{\beta}_{\user2{N}}-\varvec{\beta}_{\user2{S}}) (\varvec{\varGamma}^{\varvec{\prime}}-\varvec{\varPsi}^{\varvec{\prime}}) \user2{C}_{\varvec{\Updelta} \user2{g}}}{C}}. \end{aligned} $$

Again, note the similarity between non-social and social selection gradient and DGEs and IGEs. Both social selection and IGEs act in one direction to determine the mean fitness of a particular group, however, both the social selection gradient and IGEs act in the opposite direction when determining the fitness of an individual within its group. Thus, if the social selection gradient (or IGEs) increases overall fitness of the group, it decreases the relative fitness of an individual when compared to its social partners in the same group. Similarly, when social selection (or IGEs) decreases fitness of the group, it will act positively on the fitness of an individual within its group.

When simplified to a one-trait scenario, our inference of fitness in all four modelled scenarios is in full agreement with those provided by Bijma and Wade (2008) in Table 2, where *A*_*D*,*i*_ corresponds to our $\varGamma^\prime$ and *A*_*S*,*j*_ corresponds to $\varPsi^\prime.$ However, our multi-trait approach allows investigation of more general and complex scenarios when multiple traits interact. This is crucial in many cases, for example when a signalling trait influences behavioural responses in social partners, e.g. green-beards (Dawkins 1976). Furthermore, we show how both $\varvec{\varGamma}^{\varvec{\prime}}$ and $\varvec{\varPsi}^{\varvec{\prime}}$ can be directly determined from the interaction matrix $\varvec{\varPsi}$ in Eq. (10) (Table 1).

**Table 1:** Symbols used in the model

Symbol	Description
$\user2{g}(\user2{p})$	Column vector of individual genotype (phenotype)
*w*	Relative fitness of an individual
$\user2{e}$	Column vector of environmental effects
$\varvec{\varGamma}$	Matrix mediating the translation of an individual’s own genotype into its phenotype
$\varvec{\varPsi}$	Square matrix of phenotypic influences; $\varPsi_{k,l}$ denotes the effect of trait *l* on trait *k*
$\varvec{\varGamma}^{\varvec{\prime}}$	Matrix of direct genetic effects
$\varvec{\varPsi}^{\varvec{\prime}}$	Matrix of indirect genetic effects
$\varvec{\beta}_{\user2{N}}$	Non-social selection gradient, row vector
$\varvec{\beta}_{\user2{S}}$	Social selection gradient, row vector
*N*	Number of interacting individuals in one group
*M*	Number of groups in a population
*i*, *k*	Individual and group index
${\overline{\user2{g}}} ({\overline{\user2{p}}})$	Column vector of mean genotype (phenotype) of a particular group
$\varvec{\vartriangle} \user2{g} (\varvec{\vartriangle} \user2{p})$	Deviation of an individual’s genotype (phenotype) from mean genotypic values of the group it belongs to
${\overline{\overline{\user2{g}}}} ({\overline{\overline{\user2{p}}}})$	Vector of mean genotypic (phenotypic) values across whole population, set to 0
$\user2{C}_{\overline{g}}$	Inter-group genotypic variance-covariance matrix
$\user2{C}_{\vartriangle g}$	Intra-group genotypic variance-covariance matrix

## Discussion

The key question we address in this paper is how IGEs and social selection gradient differ in their effects on the evolution of traits involved in social interactions. We investigated under what conditions the predictions of social selection models differ from those of IGE models.

While Wolf et al. (1999) and McGlothlin et al. (2010) consider IGEs only as a factor creating covariance between phenotypes, upon which social selection can operate, we show that the effect of both IGEs and social selection on trait evolution is symmetrical. Similarly to Bijma and Wade (2008), we demonstrate that IGEs can lead to social selection even in the absence of a social selection gradient. We investigate the response to selection in all four scenarios of social interactions and its dependence on intra- and inter-group genotypic variances.

Despite their similarity, IGEs differ from social selection models in that in the former the possibility of feedback loops exists, i.e. when the phenotype of a focal individual influences phenotypes of a different individual, which may, in turn, alter the phenotype of the focal individual (Wolf et al. 1999; McGlothlin et al. 2010; Trubenová and Hager 2012). This situation cannot be modelled in the framework of social selection models. Evolutionary models using social selection gradients assume that interactions have direct consequences for the fitness of conspecifics. Often, an associated change in the social partner’s phenotype is implicitly assumed but does not capture situations, in which one trait affected by interactions may alter the same or other traits in the focal individual (feedback loops). Such feedback loops, however, may be important for the evolution of social traits. For example, Rutte and Taborsky (2007) have shown that cooperative behaviour in female rats is influenced by prior receipt of help. Rats that received help from a social partner were more likely to help their conspecifics. Again, scenarios involving such feedback loops are easily incorporated into IGE models (by populating the interaction matrix $\varvec{\varPsi}$) but are not part of social selection gradient models.

### Both IGEs and Social Selection Gradient Create Selective Pressure at the Group Level

IGEs and social selection gradients can both create selective pressure at the group level through causing fitness differentials between groups. However, under certain circumstances, IGEs may have stronger effects in creating fitness differences betweeen groups. Specifically, an individual’s fitness within a group may be more influenced by the fitness of all group members than by its own fitness. We can see from Eqs. (7), (11) and (13) that fitness of an individual depends both on its own genotype and mean genotype of its group (group properties). While the first term in these equations shows the dependence of fitness on mean group genotype, the second term describes the fitness deviation from the mean group fitness, which depends on individual genotype. Thus, while the first term enhances differences between groups, the second term describes relative fitness of an individual within its group.

**Fig. 2:**
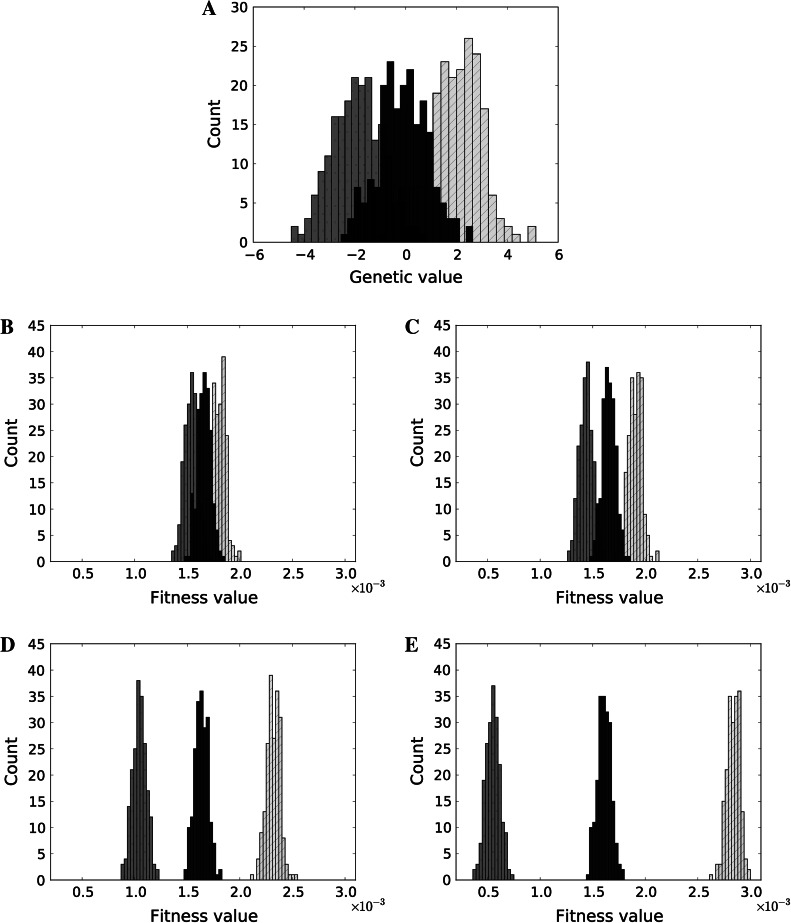
**a** Distribution of genetic values in three different groups of 200 individuals, used in the following simulations (**b**–**e**). Comparison of all four scenarios: **b** No IGEs occur and no social selection gradient exists; **c** No IGEs occur but a social selection gradient exists (β_*S*_ = 0.05); **d** IGEs occur $(\varPsi=0.05)$ but no social selection gradient exists; **e** Both IGEs and social selection gradient exist $(\varPsi=0.05, \beta_S = 0.05).$ Non-social selection is positive in all four scenarios (β_*N*_ = 1)

These equations reveal that the effect of IGEs is similar to the effect of a social selection gradient. While both IGEs and social selection gradients may decrease the relative fitness of an individual within its own group, both factors also enhance fitness differences between groups. In contrast to previous work by Wolf et al. (1999) and McGlothlin et al. (2010), but in agreement with Bijma and Wade (2008), we show that both IGEs and social selection gradients can create pressure at the group level separately by causing differences in fitness between groups (Fig. 2a–e). The response to selection in both frameworks then depends on the genotypic variance (or covariance, if genes covary) within as well as between groups.

However, IGEs may depend non-linearly on the interaction strength between phenotypes, which, under certain circumstances, can cause extreme phenotypic values or increase the phenotypic variance (Trubenová and Hager, 2012). This suggests that IGEs can be more important than a social selection gradient in determining which level of selection (the individual or group level) will have a stronger impact on trait evolution. Specifically, if the strength of interaction is close to values where $det( {\boldsymbol{I}} - N \varvec{\varPsi}+{\boldsymbol{\varPsi}}) = 0$, small differences in the mean genotype between groups may translate into substantial differences in fitness between groups. In such a case, group properties are much more important for an individual’s fitness, and the selection pressure at the level of groups becomes stronger than selection at the level of individuals. Fig. 2 shows the effect of social selection (Fig. 2c) and IGEs (Fig. 2d) (β_*S*_ = 0.05 or $\varPsi=0.05$) in groups of 200 individuals, when a trait affects its own expression in social partners. Due to the feedback that occurs in such a scenario (Trubenová and Hager, 2012), IGEs can create larger differences between groups than a social selection gradient.

When both social selection gradients and IGEs create selection pressure at the group level, both also weaken the correlation between genotype and fitness. Larger differences between groups will lead to some individuals having higher fitness than individuals in other groups despite the fact that the latter have genotypes that would result in higher fitness in the absence of the group effect. This will weaken the selection pressure at the individual level and may slow down evolution of some traits. However, it may lead to evolution of traits that would not normally be selected for under conditions of non-social selection, e.g. altruistic behaviour. On the other hand, when the strength of interaction is close to values where $det( \user2{I} +{\boldsymbol{\varPsi}}) = 0$, an individual’s own genotype is much more important for its fitness than the group mean genotype, even in the presence of a social selection gradient. In such a case, IGEs will decrease the importance of groups and can increase the correlation between fitness and genotype. This may lead to stronger selection pressure at the level of individuals, thus accelerating the rate of the evolution.

### Both social selection gradient and IGEs may change the direction of evolution

We have shown that both social selection gradients and IGEs can cause fitness differences between groups, that both can have negative effects on individual fitness within a group, and that both can determine whether the change in the mean genotype (or phenotype) will be positive or negative. Thus, both social selection gradients and IGEs may alter the expected outcome of selection and trait evolution compared to scenarios, in which no social interactions occur. This agrees with prior work on IGEs (Moore et al. 1997; Wolf et al. 1999).

**Fig. 3:**
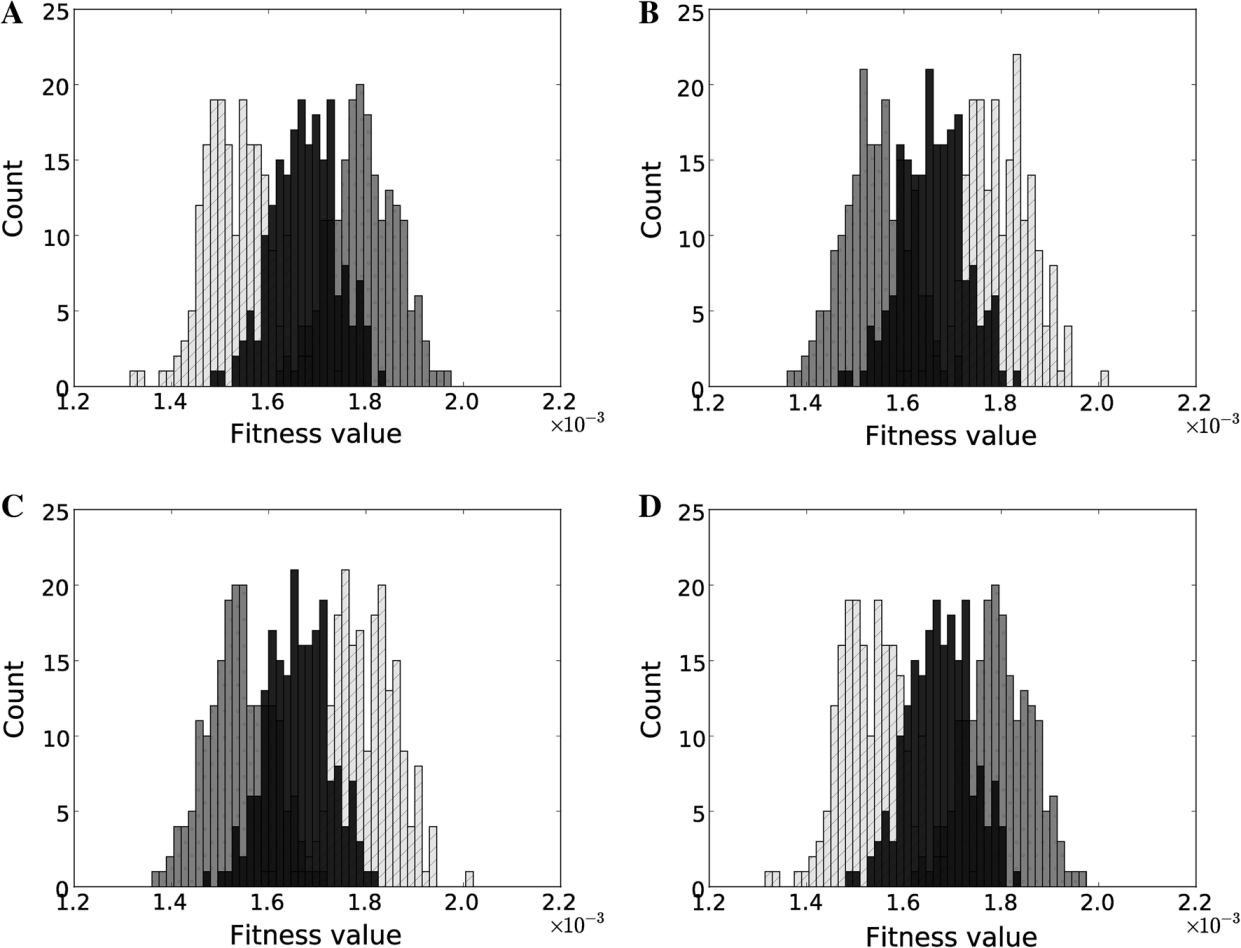
**a**–**d** IGEs and social selection gradient may change the direction of evolution: **a** Negative non-social selection gradient; **b** negative non-social selection, but positive social selection gradient present (β_*N*_ =  − 1, β_*S*_ = 0.011); **c** negative non-social selection gradient, positive IGEs present $(\varPsi = 0.01)$; **d** negative non-social selection gradient, both IGEs and social selection positive $(\beta_N = 1, \varPsi = 0.01)$

Figure 3 shows the effect of a social selection gradient and IGEs on individual fitness for all four scenarios. While the distribution of genotype values is the same in all scenarios (Fig. 2a), the distributions of fitness differ between them. For example, in Fig. 3a the group with the lowest values (displayed in dark grey) is the fittest, whereas in Fig. 3b and c the population with the highest values is the fittest (shown in light grey) because of the separate effects of social selection and IGEs. Interestingly, both types of interactions (social selection gradient and IGEs) act multiplicatively. Thus, while any of them can create differences between groups, the most pronounced effect occurs when they both work together, in the same direction (as in Fig. 2e). However, they can also cancel each other, as shown in Fig. 3d, where the population with the lowest values (dark grey) is the fittest, which is the same as when neither IGEs nor social selection gradient exist.

### Direction of Evolution May Differ Between Phenotype and Genotype

Because social interactions can change the relationship between genotype and phenotype from what is expected in the absence of interactions, the phenotypic response to selection does not have to be in the same direction as the genotypic response to selection (change in the mean genotype). All phenotypic values will depend on the structure of the new population, i.e. the intra- and inter-group genotypic variance. The mean phenotype will also change, and can be expressed as

15 $$ \vartriangle\overline{\overline{\user2{p}}} =(\varvec{\varGamma}^{\varvec{\prime}} + (N-1)\varvec{\varPsi}^{\varvec{\prime}}) \vartriangle\overline{\overline{\user2{g}}}. $$

The term $\varvec{\varGamma}^{\varvec{\prime}} + (N-1)\varvec{\varPsi}^{\varvec{\prime}},$ when negative, will cause a difference in sign between the change of the mean phenotype and mean genotype. For example, when a trait (e.g. aggression) has an effect on the same trait in social partners, any interaction strength $(\varPsi)$ greater than 1/(*N* − 1) will cause the term $\varGamma^\prime + (N-1)\varPsi^\prime$ to be negative. Therefore, the sign of the phenotypic and genotypic response to selection will be opposite. For example, the mean value of a gene associated with an altruistic trait may decrease in the population (negative genotypic response to selection) due to the higher reproductive success of non-altruistic individuals. However, following equation (15), the overall level of altruistic behaviour may increase in the population given these individuals interact in a certain way $(\varPsi>1/(N-1))$. In other words, phenotypic and genotypic evolution may move in different directions, as a consequence of IGEs and group size.

**Fig. 4:**
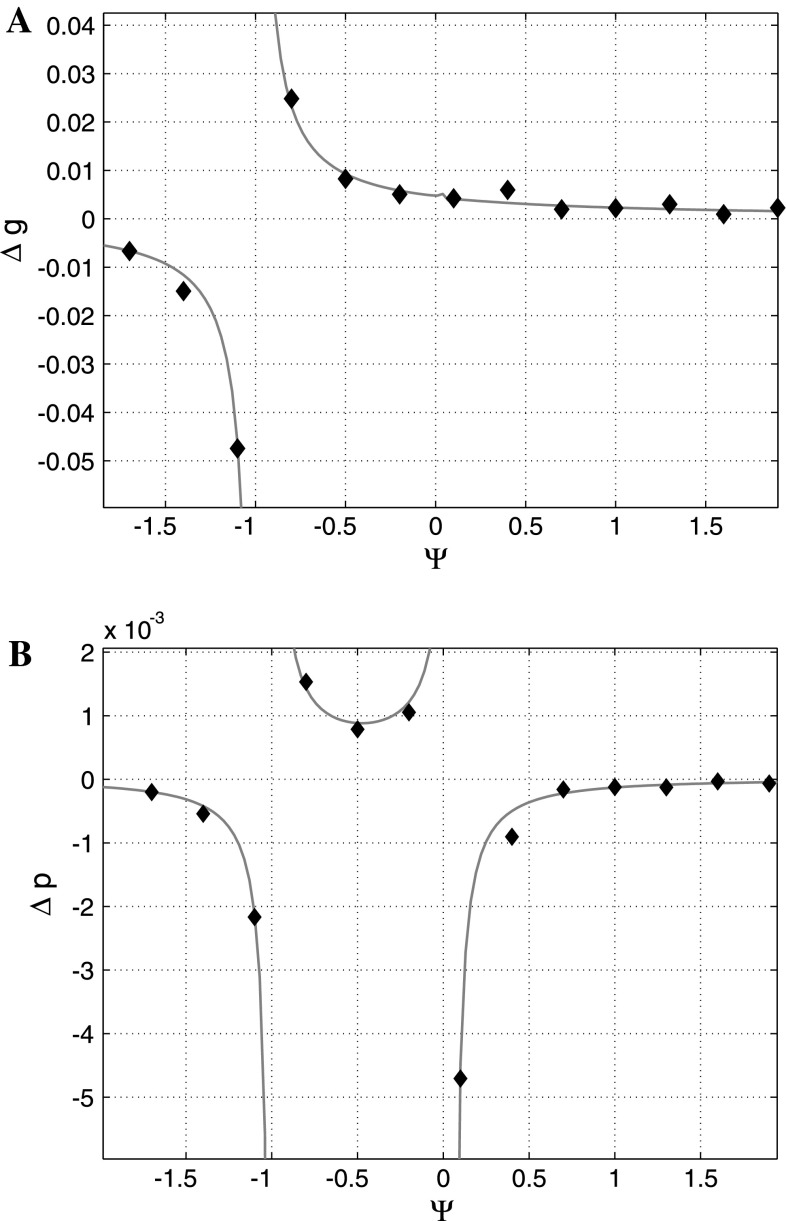
Change in genotypic $(\vartriangle\overline{\overline{\user2{g}}})$ and phenotypic $(\vartriangle\overline{\overline{\user2{p}}})$ response to selection. **a** genotypic response to selection, **b** phenotypic response to selection. While the change in genotype is positive for any interaction strength in this graph, the phenotypic response to selection is negative for $\varPsi > 1/(N-1)$. The *diamonds* show results of the simulation (mean of 500 trials), the line gives the analytical solution

Note, that this difference in directions does not depend on the selection gradients, only on the interactions leading to IGEs. Therefore, only IGEs, not social selection gradients, may cause the difference in directions. This is a key difference between IGE and social selection models. Figure 4 shows the difference between genotypic and phenotypic response to selection for a range of interaction strength values in a population of 50 groups when a given trait has an effect on the same trait in social partners. We have calculated the response to selection both analytically and via simulation of selection. Results of the simulation are calculated as a mean of 500 calculations.

We note, however, whether or not the values of $\varvec{\varPsi}$ assumed in our model occur in real populations remains to be determined empirically.

## Conclusion

In our study, we compared the effects of IGEs and social selection gradients on trait evolution in a subdivided population. We highlight the importance of IGEs and show that their effect is equivalent to that of social selection. Thus, IGEs may not only create covariances between phenotypes, on which social selection can operate, but IGEs can directly create selection pressure at the group level and lead to the evolution of social traits that would not evolve in the absence of interactions. Finally, we show that despite similarities between IGE and social selection concepts, only IGEs may lead to phenotypic and genotypic evolution moving in different directions. Therefore, the IGE framework seems more suited to modelling scenarios where social interactions lead to feedback effects between traits involved, for example cooperative behaviour.
